# Density and Conservation Optimization of the Generalized Masked-Minimizer Sketching Scheme

**DOI:** 10.1089/cmb.2023.0212

**Published:** 2024-01-11

**Authors:** Minh Hoang, Guillaume Marçais, Carl Kingsford

**Affiliations:** ^1^ Department of Computer Science, and Carnegie Mellon University, Pittsburgh, Pennsylvania, USA.; ^2^ Department of Computational Biology, Carnegie Mellon University, Pittsburgh, Pennsylvania, USA.

**Keywords:** deep learning, optimization, sequence sketching

## Abstract

Minimizers and syncmers are sketching methods that sample representative *k*-mer seeds from a long string. The minimizer scheme guarantees a well-spread *k*-mer sketch (high coverage) while seeking to minimize the sketch size (low density). The syncmer scheme yields sketches that are more robust to base substitutions (high conservation) on random sequences, but do not have the coverage guarantee of minimizers. These sketching metrics are generally adversarial to one another, especially in the context of sketch optimization for a specific sequence, and thus are difficult to be simultaneously achieved. The parameterized syncmer scheme was recently introduced as a generalization of syncmers with more flexible sampling rules and empirically better coverage than the original syncmer variants. However, no approach exists to optimize parameterized syncmers. To address this shortcoming, we introduce a new scheme called masked minimizers that generalizes minimizers in manner analogous to how parameterized syncmers generalize syncmers and allows us to extend existing optimization techniques developed for minimizers. This results in a practical algorithm to optimize the masked minimizer scheme with respect to both density and conservation. We evaluate the optimization algorithm on various benchmark genomes and show that our algorithm finds sketches that are overall more compact, well-spread, and robust to substitutions than those found by previous methods. Our implementation is released at https://github.com/Kingsford-Group/maskedminimizer. This new technique will enable more efficient and robust genomic analyses in the many settings where minimizers and syncmers are used.

## 1. INTRODUCTION

Minimizers (Roberts et al., 2005; Schleimer et al., 2003) and syncmers (Edgar, 2021) are methods to deterministically sample *k*-mers from a sequence at approximately regular intervals. These sketching methods preserve sufficient information about the sequence identity in the set of sampled *k*-mers for comparison purposes, and they are widely used to reduce run-time and memory consumption in bioinformatics programs such as read mappers (Jain et al., 2022; Li, 2018), *k*-mer counters (Deorowicz et al., 2015; Erbert et al., 2017), high-throughput sequencing (Ben-Ari et al., 2021; Nyström-Persson et al., 2021), and genome assemblers (Ekim et al., 2021).

The *k*-mer sampling minimizer scheme is derived from a *k*-mer ordering. That is, the minimizer scheme selects the lowest-ranked *k*-mer (e.g., minimizer) from each window (substring with fixed length greater than *k*) in the input sequence. While the minimizer sampling rule is dependent on other *k*-mers in the same context window, the syncmer sampling rule trades off this window sampling mechanism for other useful properties, such as better robustness when sketching homologous sequences (Edgar, 2021; Shaw and Yu, 2021).

In particular, *k*-mer sampling syncmer schemes are derived from *s*-mer orderings, where s<k. Let *k_s_* be the number of *s*-mers in each *k*-mer. The *open-syncmer* variant samples every *k*-mer in which the lowest-ranked *s*-mer is found at the $t^{th}$ offset position for some fixed $t\in \left[0,k_{s}-1\right]$. The *closed-syncmer* sampling rule sets this offset position to be either the first or the last position.

The parameterized syncmer scheme (Dutta et al., 2022) generalizes these syncmer variants using a subset parameter that encodes the selection rule. Specifically, given some subset $v\subseteq \left[0,k_{s}-1\right]$, a *v*-parameterized syncmer scheme samples every *k*-mer in which the lowest-ranked *s*-mer is found at *some offset position* in *v*.

This flexible encoding of sampling rules offers a practical handle on the performance of syncmers, where subsets that correspond to neither open-syncmer (i.e., v={t}) nor closed-syncmer (i.e., $v=\left\{0,k_{s}-1\right\}$) have been shown to outperform both original variants (Dutta et al., 2022).

The quality of the *k*-mer sketches obtained by these schemes can be quantified by various metrics. Schleimer et al. (2003) uses the *density* metric (i.e., the sketch size proportionate to the sequence length) to estimate the degree of cost savings in downstream applications. More recently, Edgar (2021) proposes the *conservation* metric (i.e., the likelihood of sketched *k*-mers to be persistently sampled across homologous sequences) and argues that high conservation is preferable when comparing sequences that might have diverged due to mutations and/or sequencing error.

Shaw and Yu (2021) subsequently demonstrated that syncmers have better expected conservation than minimizers when both the input sequence and the ordering parameter are randomly drawn from uniform distributions. In this work, we consider the *coverage* metric that measures the spread of the sketch across the input sequence. We show that these three metrics are often adversarial to one another, and consequently propose a more holistic *generalized sketch score* (GSS) to evaluate sketching performance (Section 3).

Previous studies have established expected density guarantees for minimizers (Marçais et al., 2017; Schleimer et al., 2003) and syncmers (Edgar, 2021) with uniformly random input sequences. These results support the use of a fixed ordering for general sketching applications. However, when dealing with scenarios involving multiple query sequences being aligned against a single reference string, such as genome assembly, it is often more desirable to have an ordering that is optimally configured based on the reference.

For instance, Zheng et al. (2021) and Hoang et al. (2022a) have developed practical algorithms to optimize the *k*-mer ordering in the minimizer method. These studies have demonstrated that sequence-specific minimizer sketches generally achieve much lower density compared with non-optimized minimizer sketches.

Nonetheless, these optimization methods cannot be directly applied to configure low-density syncmer sketches, because they explicitly leverage the minimizer window sampling mechanism to construct their respective learning objectives. For example, the polar set method adopts a heuristic that selects as many *k*-mers as possible from the set of *k*-mers that are *w* (i.e., window size) bases apart (Zheng et al., 2021), whereas the DeepMinimizer method constructs a sinusoidal template function with period *w* to guide optimization (Hoang et al., 2022a).

In addition, syncmers have no minimum density guarantee, unlike minimizers that derive this property from the window sampling mechanism. As such, optimizing the syncmer method for a specific sequence can potentially result in a vacuous sketch with zero coverage (Section 8.1). Finally, extending previous density optimization methods to account for the conservation metric is also challenging, as conservation and density are adversarial metrics (Section 3.4).

To address these challenges, we adapt the parameterized syncmer framework (Dutta et al., 2022) such that the pattern-aware sampling rules are applied in conjunction with the window sampling rule of minimizers. We call this adaptation masked minimizers. Specifically, given a subset *v* (or equivalently a binary mask variable in our formulation), the masked minimizer scheme selects all minimizers that are found at some offset position in *v* (with respect to the windows they minimizer).

Similar to the parameterized syncmer framework, the pattern-aware sampling rules give masked minimizers the ability to balance the trade-off between density, conservation, and coverage. However, our formulation differs from that of Dutta et al. (2022) since the selection of a masked minimizer depends on *k*-mers around it, whereas a parameterized syncmer is selected in a context-free manner. This distinction is identical to how minimizers and syncmers differ and allows us to leverage and extend density optimization algorithms developed for minimizers (Section 5).

In particular, we develop a sequence-specific optimization algorithm for masked minimizers that extends the DeepMinimizer method (Hoang et al., 2022a). Our algorithm adopts a bi-level learning framework that alternates between pruning the mask variable and learning the *k*-mer ordering. Given a fixed mask, the inner loop optimizes the ordering via combining two differentiable objectives that respectively surrogate the density and conservation of the masked minimizer scheme. Alternately, the outer loop searches for the optimal mask via greedily pruning its set bits, suggesting pruned candidates to the inner loop, and selecting one that yields the best metric gain.

We show that the optimized masked minimizer sketch of various human and bacterial genomes consistently achieves better GSS than previous optimization approaches, such as Miniception (Zheng et al., 2020), PASHA (Ekim et al., 2020), and DeepMinimizer (Hong et al., 2022a).

We also discover a specific class of complement mask patterns (i.e., masks that include most offset positions except one) that combines desirable properties from minimizers (i.e., high coverage) and open-syncmers (i.e., tolerance to low-complexity sequences).

In summary, our contributions include: (1) an adaptation of the parameterized syncmer method (Dutta et al., 2022) that generalizes minimizers, which we call masked minimizers, (2) a novel sketching metric that combines and reflects the trade-off among density, conservation, and coverage, which we call the GSS, and (3) a bi-level optimization algorithm for the masked minimizer scheme, which jointly selects the optimal mask and ordering with respect to the GSS metric.

Our sequence-specific sketching framework combines the strength of sequence-specific minimizers and parameterized syncmers to improve sketching performance in a holistic manner.

## 2. SUBSTRING SAMPLING SCHEMES

### 2.1. Notation

Let Σ be an arbitrary alphabet over which an input sequence $S\in \Sigma^{L}$ is defined. We further let $\kappa_{i}^{k}$ and $L_{k}=\Delta L-k+1$, respectively, denote the $i^{th}$ *k*-mer and the total number of overlapping *k*-mers in *S*. A (w,k)-window is a substring of length $w_{k}=\Delta w+k-1$ and contains exactly *w* overlapping *k*-mers. By extension, the $i^{th}$ (w,k)-window and the total number of (w,k)-windows are denoted by $\kappa_{i}^{w_{k}}$ and $L_{w_{k}}=L-w_{k}+1$.

Finally, given an arbitrary *k*-mer scoring function $f:\Sigma^{k}\to \left[0,1\right]$, we define an index selector function mf(a,b) =Δ argminj∈[0,b−1]f(κa+jk), which helps to define the various *k*-mer sketching schemes.

### 2.2. Minimizers

The *k*-mer sampling minimizer scheme is characterized by a tuple of parameters (w,k,π). Traditionally, π denotes a total ordering over the set of all *k*-mers. Equivalently, Hoang et al. (2022a) interprets this ordering as a *k*-mer scoring function $f_{\pi }:\Sigma^{k}\to \left[0,1\right]$, such that for every pair of *k*-mers $\kappa ,\kappa \prime \in \Sigma^{k}$:

(1) $$\kappa {≺}_{\pi }\kappa \prime ⇐f_{\pi }\left(\kappa \right)<f_{\pi }\left(\kappa \prime \right),$$

where ${≺}_{\pi }$ denotes the precedence of ordering in π, and tie-breaking of *k*-mers with the same score is determined by their order of appearance in the window. The minimizer method samples and reports the indices of the lowest-scoring *k*-mers (e.g., minimizers) from each (w,k)-window in *S*:

(2) $$ℳ\left(S;w,k,\pi \right)=\Delta {\left\{i+m_{f_{\pi }}\left(i,w\right)\right\}}_{i\in \left[1,L_{w_{k}}\right]}.$$

### 2.3. Open syncmers

The *k*-mer sampling open-syncmer scheme (Edgar, 2021) is specified by a tuple (k,s,t,π). Here, the parameter s<k implicitly characterizes the representation of *k*-mers as the collection of their constituent *s*-mers. We additionally denote the number of *s*-mers in each *k*-mer by $k_{s}=\Delta k-s+1$. The parameter π denotes a total ordering over the set of *s*-mers and can likewise be represented by a scoring function $f_{\pi }:\Sigma^{s}\to \left[0,1\right]$.

Finally, the *qualifying offset position* 0≤t≤k−1 indicates that the scheme will sample all *k*-mers in which the lowest-scoring constituent *s*-mer is exactly at position *t* (relative to the *k*-mer position):

(3) $$O\left(S;k,s,t,\pi \right)=\Delta {\left\{i|m_{f_{\pi }}\left(i,k_{s}\right)=t\right\}}_{i\in \left[1,L_{k}\right]}.$$

### 2.4. Parameterized syncmers

Based on the syncmer concept, Dutta et al. (2022) introduces the parameterized syncmer scheme, which replaces *t* by a subset of qualifying offset positions $v\subseteq \left[0,k_{s}-1\right]$. The parameterized syncmer method samples and reports the indices of all *k*-mers such that their lowest-scoring constituent *s*-mers are found at some offset positions in *v*:

(4) $$O^{+}\left(S;k,s,v,\pi \right)=\Delta {\left\{i|m_{f_{\pi }}\left(i,k_{s}\right)\in v\right\}}_{i\in \left[1,L_{k}\right]}.$$

Setting |v|=1 and $v=\left\{0,k_{s}-1\right\}$, respectively, recovers the open syncmer scheme above and the closed syncmer scheme (Edgar, 2021).

### 2.5. Masked minimizers

While sequence-specific optimization of minimizers with respect to the density metric has been well addressed (Ekim et al., 2020; Hoang et al., 2022a; Zheng et al., 2020), the same capability has not been developed for either open syncmers, parameterized syncmers, or for other metrics than density.

To overcome this challenge, our goal is to extend the minimizer method with pattern-based sampling rules similar to that of parameterized syncmers. This extension allows us to incorporate desirable properties from the syncmer family, yet fully retain access to density optimization algorithms developed for minimizers.

To this end, we introduce the masked minimizer scheme specified by the tuple (w,k,v,π). The parameters w,k,π maintain the same definition as in the original minimizer scheme. Similar to parameterized syncmers (Dutta et al., 2022), *v* denotes a subset of qualifying offsets (e.g., a binary mask) such that a minimizer is chosen only if its relative location in the window is within *v*. The masked minimizer sampling rule is:

(5) $$V\left(S;w,k,v,\pi \right)=\Delta {\left\{i+m_{f_{\pi }}\left(i,w\right)|m_{f_{\pi }}\left(i,w\right)\in v\right\}}_{i\in \left[1,L_{w_{k}}\right]}.$$

## 3. SKETCHING METRICS

### 3.1. Density

Let X be an arbitrary *k*-mer sampling scheme parameterized by θ. The density metric (Marçais et al., 2017) measures the size of the sketch X(S;θ) relative to the number of *k*-mer in *S* (lower is better):

(6) $$D\left(S;X,\theta \right)=\Delta \frac{1}{L_{k}}|X\left(S;\theta \right)|.$$

### 3.2. Conservation

Let S′ be a homologous sequence to *S* (e.g., differing by a few random base substitutions), and suppose S′ follows some arbitrary distribution *p_S_*. The conservation metric (Edgar, 2021) measures the expected number of *bases* that are present in both X(S;θ) and X(S′;θ), relative to the number of *k*-mers in *S* (higher is better). For ease of comparison to the density metric, we instead define the conservation metric in terms of the number of sketched *k*-mers:

(7) $$C\left(S;X,\theta \right)=\Delta \frac{1}{L_{k}}{ℰ}_{S\prime ∼p_{S}}\left|X\left(S;\theta \right)\cap X\left(S\prime ;\theta \right)\right|.$$

### 3.3. Coverage

When too few *k*-mers are selected, the sketch will not sufficiently cover the sequence and is therefore not useful in practice. Although the minimizer scheme ensures that every (w,k)-window will overlap at least one sampled *k*-mer, no such guarantee exists for masked minimizers or parameterized syncmers in general.

For example, an open syncmer scheme with offset *t* can theoretically select an empty sketch if the lowest scoring *s*-mer is always found within the first t−1 offset positions. Our empirical study further shows that such a vacuous sketch can be obtained simply via optimizing for low density and high conservation (Section 8.1).

To formally quantify this property, we introduce the notion of *w*-coverage. The *w*-coverage metric computes the fraction of (w,k)-windows that overlap at least one sampled *k*-mer in X(S;θ). This means a minimizer sketch is guaranteed to have a *w*-coverage value of 1 by construction, whereas an empty sketch has a *w*-coverage value of 0. The *w*-coverage metric is formally given by:




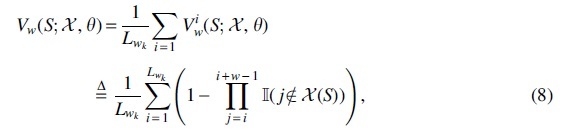




where $V_{w}^{i}$ indicates the event $\kappa_{i}^{w_{k}}$ overlaps at least one sampled location in X(S).

### 3.4. Generalized sketch score

It is straight-forward to see that:

$$C\left(S;X,\theta \right)=\frac{1}{L}{ℰ}_{S\prime ∼p_{S}}\left|X\left(S;\theta \right)\cap X\left(S\prime ;\theta \right)\right|$$

$$\leq \frac{1}{L}{ℰ}_{S\prime ∼p_{S}}\left|X\left(S;\theta \right)\right|$$

(9) $$=\frac{1}{L}\left|X\left(S;\theta \right)\right|=D\left(S;X,\theta \right).$$

The above derivation implies that conservation is upper-bounded by density for any arbitrary sketching scheme X. Thus, individually optimizing for density or conservation will likely worsen the other. Since these metrics are mutually conflicting and do not quantify coverage, neither can sufficiently measure the quality of a sketch. This motivates us to construct a more holistic sketching metric, which we call the GSS, to evaluate the performance of sketching schemes.

Intuitively, the GSS metric encourages striking a balance between high conservation, low density, and high coverage. This is achieved by measuring the trade-off ratio between conservation/density, and normalizing this value by the *w*-coverage score of the sketch:

(10) $$G_{w}\left(S;X,\theta \right)=\Delta \frac{C\left(S;X,\theta \right)}{D\left(S;X,\theta \right)}\times V_{w}\left(S;X,\theta \right).$$

As a consequence of Eqs. (8) and (9), *G_w_* is guaranteed to be in [0,1].

## 4. ANALYSIS

This section provides an analysis of the change in performance of the masked minimizer scheme as *v* varies in the power set of [0,w−1]. In particular, we ask whether conservation/density will improve with more or fewer offset locations in the qualifying subset *v*? Specifically, let θ=(w,k,v,π) and θ′=(w,k,v′,π) be the parameters defining two masked minimizer schemes such that v⊆v′⊆[0,w−1]; our analysis seeks to bound their performance gap in terms of density and conservation metrics.

**Proposition 1.** *For any input sequence S and parameters* θ,θ′ *defined above, we have* V(S;θ)⊆V(S;θ′).

*Proof.* Let i∈V(S;θ). By definition of the masked minimizer sampling rule, we know that there exists $j\in \left[1,L_{w_{k}}\right]$ such that $j+m_{f_{\pi }}\left(j,w\right)=i$ and $m_{f_{\pi }}\left(j,w\right)\in v$. Since v⊆v′, we also have $m_{f_{\pi }}\left(j,w\right)\in v\prime$, which implies i∈V(S;θ′), again by definition of the masked minimizer rule. Therefore, V(S;θ)⊆V(S;θ′).

**Corollary 1** (Density gap). *For any input sequence S and parameters* θ,θ′ *defined above, we have* D(S;V,θ)≤D(S;V,θ′).

*Proof.* By definition of density:

(11) $$D\left(S;V,\theta \right)=\frac{\left|V\left(S;\theta \right)\right|}{L_{k}}\leq \frac{\left|V\left(S;\theta \prime \right)\right|}{L_{k}}=D\left(S;V,\theta \prime \right),$$

where the inequality follows directly from Proposition 1.

**Corollary 2** (Conservation gap). *For any input sequence S and parameters* θ,θ′ *defined above, we have* C(S;V,θ)≤C(S;V,θ′).

*Proof.* Let S′ be a homologous copy of *S* obtained through simulating base substitutions. We additionally define $\alpha_{i}\left(S\prime ,\theta^{†}\right)=\Delta ℐ\left(i\in V\left(S;\theta^{†}\right)\cap V\left(S\prime ;\theta^{†}\right)\right)$, which indicates the event that *i* is preserved in both $V\left(S;\theta^{†}\right)$ and $V\left(S;\theta^{†}\right)$ for some arbitrary sampling parameter tuple $\theta^{†}$. We then have the following:

$$\alpha_{i}\left(S\prime ,\theta \right)=ℐ\left(i\in V\left(S;\theta \right)\right)\times ℐ\left(i\in V\left(S\prime ;\theta \right)\right)$$

≤ℐ(i∈V(S;θ′))×ℐ(i∈V(S′;θ′))

(12) $$=\alpha_{i}\left(S\prime ,\theta \prime \right),$$

where the inequality follows from Proposition 1, and the fact that the indicator variables take values in {0,1}. We now bound the conservation gap as follows:

$$C\left(S;V,\theta \right)-C\left(S;V,\theta \prime \right)={ℰ}_{S\prime }\frac{\left|V\left(S;\theta \right)\cap V\left(S\prime ;\theta \right)\right|-\left|V\left(S;\theta \prime \right)\cap V\left(S\prime ;\theta \prime \right)\right|}{L_{k}}$$

(13) $$={ℰ}_{S\prime }\frac{\sum_{i=1}^{L_{k}}\alpha_{i}\left(S\prime ,\theta \right)-\alpha_{i}\left(S\prime ,\theta \prime \right)}{L_{k}}\leq 0,$$

where the inequality follows from Eq. (12) and linearity of expectation. Rearranging the above result concludes the proof.

These results imply that any masked minimizer scheme can improve conservation by adding more locations to its qualifying subset, or improve density by removing locations. However, as density upper-bounds conservation (Section 3.4), it is difficult to simultaneously improve both metrics by varying the mask, and hence it is necessary to formulate the optimization in terms of their trade-off ratio (e.g., the GSS metric), and with respect to the mask variable.

## 5. OPTIMIZING MASKED MINIMIZERS

**Table:** 

**Algorithm 1. Masked Minimizer Optimization**
best−gss←0
$mask\leftarrow 1^{w}$
$gss\leftarrow eval\left({\mathrm{argmin}}_{f_{\pi },g}{ℒ}_{gss}\left(seq;mask\right)\right)$ {Eq. 15}
whilegss>best−gssandnot−empty(mask)do
best−gss←gss
best−mask←mask
foroffset∈maskdo
$trial-gss\leftarrow eval\left({\mathrm{argmin}}_{f_{\pi },g}{ℒ}_{gss}\left(seq;prune\left(mask,offset\right)\right)\right)$ {Eq. 15}
iftrial−gss>gssthen
gss←trial−gss
best−mask←prune(mask,offset)
endif
endfor
mask←best−mask
endwhile
$returnf_{\pi },g,best-mask$

Given a choice of w,k, we seek to optimize the scoring function $f_{\pi }:\Sigma^{k}\to \left[0,1\right]$ and mask *v* of the masked minimizer scheme with respect to the GSS metric. To achieve this, we adopt a bi-level optimization framework, which iterates between: (1) taking gradient descent steps on the weights of $f_{\pi }$ given a fixed *v*; and (2) greedily pruning *v* to improve GSS given an optimized $f_{\pi }$. The pseudocode of our framework is given in Algorithm 1.

Our greedy pruning step (outer loop) starts with the complete qualifying set v=[0,w−1] and iteratively removes locations one by one from *v* to yield the best GSS improvement, given one full inner loop optimization of $f_{\pi }$. This outer loop terminates when no further improvement can be obtained or the mask is empty (i.e., |v|=0).

To address the inner loop optimization, we construct a differentiable loss function that extends the DeepMinimizer algorithm (Hoang et al., 2022a). As the DeepMinimizer loss function is only designed to minimize density, we augment it with an auxiliary term that aims at estimating conservation. This combined loss function surrogates the trade-off between density and conservation, and thus it will implicitly allow us to maximize the GSS metric. We describe the components of our loss function as follows:

### 5.1. Density optimization

Following Hoang et al. (2022a), we employ a pair of collaborating neural networks to model a hash function with low density on *S*:

The first neural network, PriorityNet, computes the score vector 
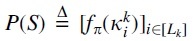
 and implicitly defines $f_{\pi }$. Due to the one-to-one design of $f_{\pi }$, P(S) recovers a total ordering, and hence a proper masked minimizer sketch (e.g., the sampling mechanism is consistent across all windows).

The second neural network, TemplateNet, computes the template vector 
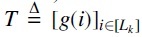
 and implicitly defines a *positional k*-mer scoring function g:ℕ→[0,1]. Hoang et al. (2022a) gives a construction of *g* such that the minimizer sketch derived from this template (via applying the minimizer sampling rule parameterized by *g*) has approximately optimal minimizer density (e.g., 1∕w). Nonetheless, this template sketch is insensitive to simple translation of identical windows, hence it is not directly useful in real alignment tasks.

Intuitively, these networks, respectively, ensure the validity of a minimizer scheme and the ideal low density. A low-density sketch, thus, can be viewed as a consensus sketch P(S) that minimizes some distance metric to an arbitrary template sketch *T* in the output space of the TemplateNet. In particular, we define this distance as follows:




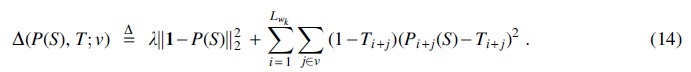




The first term $\lambda ∥1-P\left(S\right){∥}_{2}^{2}$ in Eq. (14), which follows the formulation of Hoang et al. (2022a), is a regularization term that ensures both P(S) and *T* do not trivially set *k*-mer scores to 0 to minimize their distance. The hyper-parameter λ serves as a trade-off constant between the two objective terms.

The second term differs from that of Hoang et al. (2022a) by the introduction of the inner summation over the offset positions in *v*, which is specific to the masked minimizer method. This sum represents an aggregation of weighted $\ell_{2}$-distances over all (w,k)-windows of *k*-mer scores in *P* and *T*.

The weight at each *k*-mer location is, therefore, jointly determined by its template value (i.e., how likely it is that this position will contribute to the sketch) and whether it can be found in the qualifying subset of some window (i.e., how relevant this is positioned to the current sampling rule).

### 5.2. Conservation optimization

The above objective only focuses on minimizing density. To account for conservation, our loss function ${ℒ}_{gss}$ extends Δ with an additional objective:




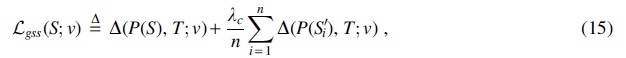




where $S\prime_{i=1\ldots n}$ denotes homologous copies of *S* randomly drawn from *p_S_*, and $\lambda_{c}$ balances between the density and conservation objectives. ${ℒ}_{gss}$ is optimized with respect to the combined parameters of $f_{\pi }$ and *g*.

The first term of ${ℒ}_{gss}$ is exactly the density loss described above. The second term surrogates the conservation metric by estimating the expected Δ-distance from each $P\left(S\prime_{i}\right)$ to the template *T*. When this term is small, we intuitively expect ${\left\{P\left(S\prime_{i}\right)\right\}}_{i=1\ldots n}$ to be concentrated around *T*, and by extension P(S), as *T* is brought close to P(S) via minimizing the first term. Since the score vectors induce the sketch of $S\prime_{i}$ and *S*, this implies that the sketch of *S* is likely preserved across homologous sequences and yields high conservation.

### 5.3. Remark

Although we do not have direct results on the NP-hardness of optimizing GSS with respect to a target sequence, many problems adjacent to it, such as finding a sequence-specific universal hitting set (UHS; Orenstein et al., 2017) or the smallest size polar set (Zheng et al., 2021), have been shown to be NP-hard. A direct, brute-force strategy that performs the inner loop optimization on every possible mask would scale exponentially with the inner loop cost, and thus is prohibitively expensive.

Our pruning heuristic instead enables a worst case complexity of $O\left(w^{2}\right)$ in terms of the inner loop cost (e.g., the cost of performing the inner loop w−t times for every $t^{th}$ pruning layer).

## 6. EMPIRICAL RESULTS

In this section, we demonstrate the effectiveness of our optimization algorithm in learning high conservation, low density, and high coverage masked minimizer sketches. We also explore various ablation scenarios to confirm the practical usage of various specific masks (qualifying subsets).

### 6.1. Experimentation details

We compare the following baselines to construct the *k*-mer ordering for masked minimizers: (1) random ordering; (2) training with variants of our objective, including the DeepMinimizer loss function (Hoang et al., 2022a); (3) Miniception (Zheng et al., 2020); and (4) PASHA (Ekim et al., 2020).

All experiments are conducted on the human chromosome 1 (labeled Chr1); the centromere region of human chromosome X (labeled ChrXC); and several bacterial genomes that were previously used in Edgar (2021; labeled BTR1, BTR2, BTR3, and BTR4).

The details of these sequences are given in Section 8.2. The gradient-based loss functions are computed per batch of sampled subsequences since it is not possible to fit the entire sequence on GPU memory. Our PyTorch implementation is available at https://github.com/Kingsford-Group/maskedminimizer. Other implementation details are given in Section 8.2.

### 6.2. Adversarial relationship of density and conservation

This experiment demonstrates that density and conservation are, indeed, conflicting objectives and confirms our argument in Section 3. Specifically, we train two masked minimizers using the minimizer mask $v_{m}=\left[0,w-1\right]$ and the open-syncmer mask $v_{o}=\left\{w∕2\right\}$ for w=7,k=15.

We note that the masked minimizer scheme with *v_o_* employs the window-based sampling mechanism, hence it does not recover exactly the open-syncmer scheme (or equivalently the parameterized syncmer scheme with mask *v_o_*), and only emulates its sampling pattern in the context of minimizers. We, respectively, denote these schemes by ℳ and $O_{w∕2}$ and optimize them with three variants of our loss function:

- The vanilla DeepMinimizer density loss, given by ${ℒ}_{DM}=\Delta \Delta \left(P,T\right)$.
- The conservation loss given by the second term in Eq. (15),

$${ℒ}_{con}=\Delta \frac{1}{n}\sum_{i=1}^{n}\Delta \left(P_{i},T\right).$$

- Our loss function ${ℒ}_{gss}=\Delta {ℒ}_{DM}+\lambda_{c}{ℒ}_{con}$ given in Eq. (15) with $\lambda_{c}=1$.

**FIG. 1.**
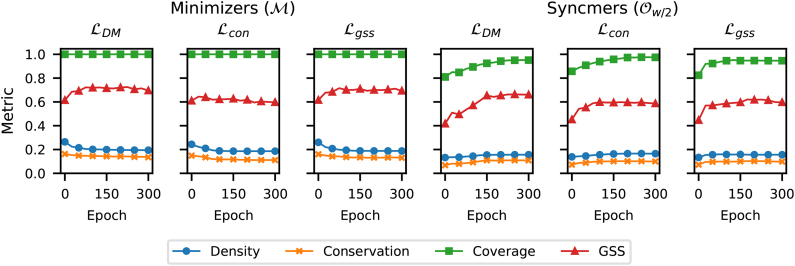
Comparing density, conservation, coverage, and GSS versus number of training epochs using different training losses and masks *v* on the bacterial genome Btr1. GSS, generalized sketch score.

Figure 1 plots the density, conservation, coverage, and GSS metrics on the sequence Btr1 across 300 training epochs for each loss function. As predicted in Section 3, we observe that the conservation metric is consistently upper-bounded by the density metric in all experiments.

In addition, we observe that neither the density nor conservation metric reflects the drop in coverage when moving from the minimizer mask ℳ to the open-syncmer mask $O_{w∕2}$. The GSS metric, on the other hand, properly reflects this by applying a discount to the performance of the open-syncmer mask.

### 6.3. Training-masked minimizers improves GSS

This section demonstrates that our loss function ${ℒ}_{gss}$ learns robustly and improves GSS in various settings of w,k, and different masks *v*. Specifically, we compare the minimizer mask (*v_m_*) and the open-syncmer mask (*v_o_*) defined above with the complement mask $v_{c}=v_{m}\setminus v_{o}$ that combines desirable properties from minimizer (e.g., high coverage) and open-syncmer mask (e.g., preventing repeated sampling in homopolymers).

We denote the complement mask by $C_{w∕2}$. Again, we do not employ the outer loop of our algorithm to search for the optimal mask since it involves multiple inner iterations of training and cannot be plotted on the same scale with other benchmarks (e.g., each corresponds to a single inner loop with 600 epochs). The performance of this outer loop training will be demonstrated in the next experiment.

**FIG. 2.**
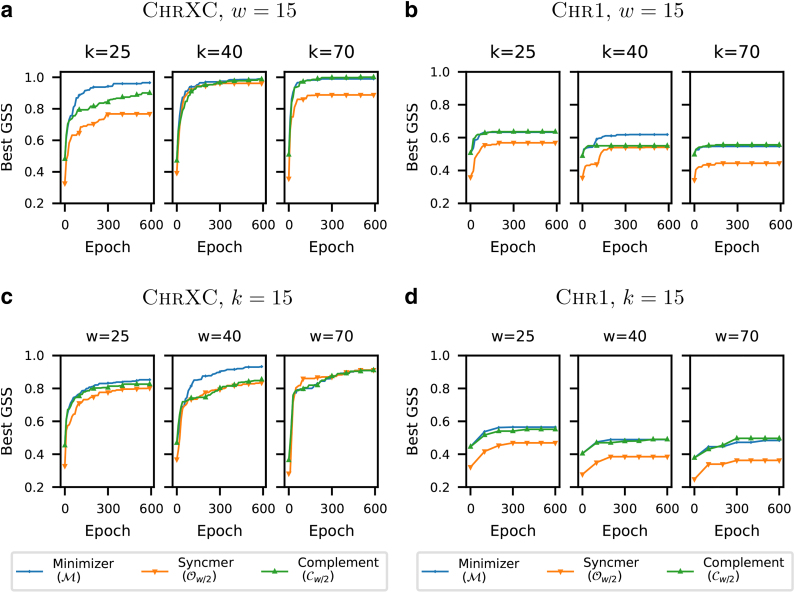
Comparing GSS of different masked minimizer variants versus number of training epochs on ChrXC and Chr1.

Figure 2 plots the GSS of the masked minimizers ℳ, $O_{w∕2}$, and $C_{w∕2}$ over 600 training epochs in two settings: (1) w=15 and k∈[25,40,70]; (2) k=15 and w∈[25,40,70]. This experiment is repeated on two sequences, ChrXC and Chr1. All experiments show that GSS steadily increases over 600 training epochs by 1.5 to 5 times that of their initial random weights.

We observe that the performance of the minimizer mask (ℳ) is highly similar to the complement mask ($C_{w∕2}$), except for (w,k)=(15,40) with Chr1 and (w,k)=(15,25),(40,15) with ChrXC. This is expected because their masks only differ by one location.

We further observe that both the minimizer mask (ℳ) and the complement mask ($C_{w∕2}$) outperform the open syncmer mask ($O_{w∕2}$) in most settings. This is most likely due to the worse coverage of open-syncmers, which has been previously observed in Figure 1. In Section 8.1, we further show the individual effects of training on the conservation and density metrics for the experiments in Figure 2a, thus confirming our analysis in Section 4.

### 6.4. Comparing GSS of different training losses and masks

In this section, we demonstrate the importance of optimizing for the mask variable. Specifically, we compare the GSS performance among methods that optimize for the *k*-mer ordering alone with respect to some fixed mask, and our method (Algorithm 1) that jointly optimizes both variables.

We, respectively, denote the optimized mask and its induced masked minimizer scheme by $v_{*}$ and V. We benchmark the performance of this mask-optimized scheme against the minimizer (ℳ), open-syncmer $\left(O_{w∕2}\right)$ and complement masks $\left(C_{w∕2}\right)$ across various optimization strategies, including random orderings, PASHA (Ekim et al., 2020), Miniception (Zheng et al., 2020), AdaOrder (Flomin et al., 2022), and gradient-based optimization with 3 different loss functions previously introduced (i.e., ${ℒ}_{DM}$, ${ℒ}_{con}$, ${ℒ}_{gss}$).

**FIG. 3.**
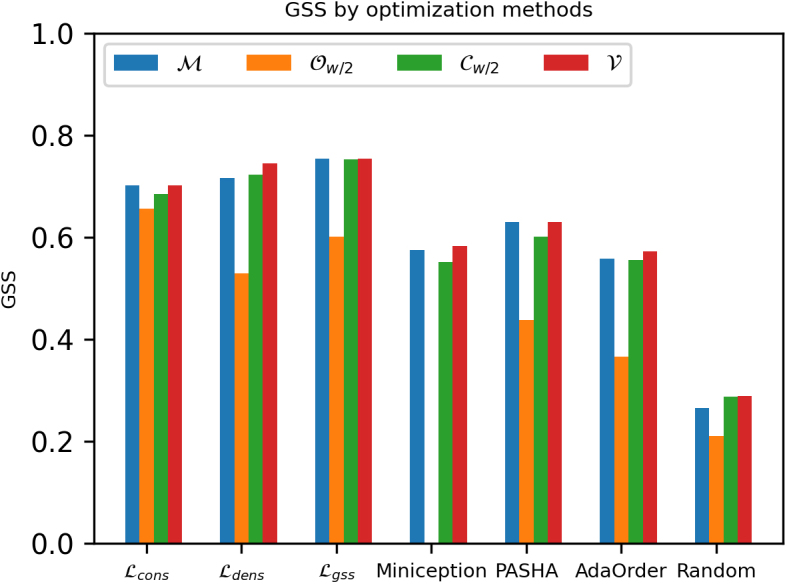
Comparing GSS of different masked minimizers using various optimization methods with w=10,k=10 on ChrXC. We, respectively, denote the minimizer mask, the open-syncmer mask, the complement mask, and the optimized mask by $ℳ,O_{w∕2},C_{w∕2},V$.

**FIG. 4.**
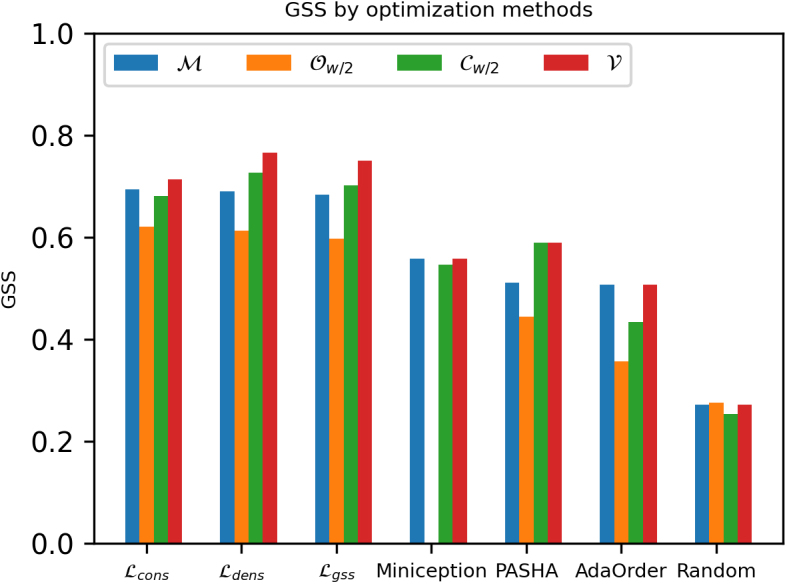
Comparing GSS of different masked minimizers using various optimization methods with w=15,k=10 on ChrXC. We, respectively, denote the minimizer mask, the open-syncmer mask, the complement mask, and the optimized mask by $ℳ,O_{w∕2},C_{w∕2},V$. PASHA.

For random ordering and the UHS-based methods, which only select the ordering once, the inner-loop optimization is simply replaced by evaluating the GSS metric with respect to the current *v*. We repeat our experiment for w=10,k=10 (Fig. 3) and w=15,k=10 (Fig. 4). The complete result tables for all combinations of w∈{10,15,20} and k∈{10,15} are reported in Section 8.1.

Among different masks of the same optimization method, we observe that the optimized mask V achieves the best GSS most frequently (i.e., 13 out of 14 scenarios). Out of 13 occurrences, V recovers the same GSS as the minimizer mask ℳ 5 times, and the same GSS as the complement mask $C_{w∕2}$ 3 times. The open-syncmer mask $O_{w∕2}$ only outperforms V one time on the random ordering baseline, with negligible margin.

Interestingly, when combined with the Miniception method, the open-syncmer mask yields 0.0 GSS, which suggests that there are no *k*-mers that can meet the sampling rule based on the ordering found by Miniception.

Among the best performing masks found by our optimization routine (Section 8.1.3), we observe that there is no fixed mask that consistently performs the best across all experiments. In addition, the maximum pruning depth observed is 3 (e.g., the algorithm terminates after 3 iterations of the outer loop because no possible GSS improvement can be found), which implies that dense masks are generally better for our benchmark sequences.

In contrast, the best performing masks reported by Dutta et al. (2022) are significantly sparser, such as v={3,9} and v={6} for k=15. We remark that this does not contradict our findings, as it was obtained on random sequences and Dutta et al. (2022) compare parameterized syncmers by the root mean squared gap lengths metric.

### 6.5. The complete mask is a good initialization

**FIG. 5.**
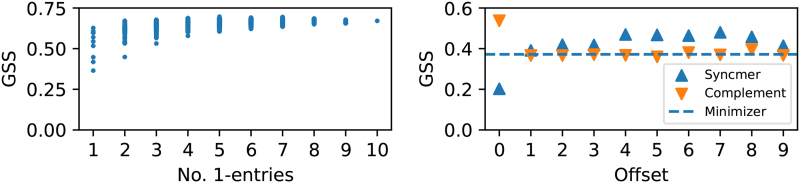
Left: GSS versus number of 1-entries of all masked minimizers trained on the bacterial genome Btr4; Right: GSS versus the offset position of various open-syncmer masks, and their complement schemes on a synthetic sequence with high homopolymer content.

We visualize the distribution of GSS across different masks. Figure 5 (left) shows the scatter plot of all $2^{w}-1$ masked minimizer schemes trained on Btr4 using ${ℒ}_{gss}$ with w=10 and k=15, grouped by the cardinality of *v*. Similar experiments on Btr1, Btr2, and Btr3 are deferred to Section 8.1. We observe that the average GSS generally increases with |v| in all experiments, which implies that the minimizer mask is a good default choice.

### 6.6. Repeated sampling in homopolymer-rich sequences

One advantage of open-syncmers with t>1 is the ability to avoid repeated sampling of identical *k*-mers in homopolymer substrings (i.e., substrings with repeated submer patterns; Edgar, 2021). To confirm this, Figure 5 (right) plots the GSS of all syncmer masks (with offsets in [0,w−1]) and their complement masks on a synthetic sequence with L=100,000 and 0.2% homopolymer content. The dotted line shows the GSS of the minimizer mask, which expectedly performs worse than most open-syncmer masks (except for v={0}) due to the repeated sampling pitfall.

In particular, because of the left-most tie breaking rule, every scheme whose mask contains the offset 0 (e.g., the minimizer mask, the open-syncmer mask with v={0}, and all complement masks except the one where 0∉v) suffers from high density. In contrast, we observe that the complement scheme with v=[1,w−1] achieves the best GSS (0.56). This is because it avoids the repeated sampling pitfall in the same way any open-syncmer scheme with t>0 does, but otherwise performs like a minimizer scheme and does not suffer from the low coverage of syncmers.

## 7. CONCLUSION

We study the masked minimizer sketching scheme that applies the parameterized syncmer sampling rules (Dutta et al., 2022) to the window sampling mechanism of minimizers. We develop a bi-level optimization framework to design masked minimizers for a specific reference sequence. To account for the conflicting sketching metrics (e.g., density, conservation, and coverage), we propose a new sketching metric called GSS.

We show that our algorithm finds combinations of masks and *k*-mer orderings that induce masked minimizer schemes with better GSS than other sketch construction methods. We additionally introduce a special category of complement masks that combine desirable properties of minimizers and syncmers. We demonstrate the robustness of these masks in both the standard setting and sketching a homopolymer-rich sequence that is known to be a pitfall for the minimizer method.

This research opens up new directions for systematic construction of sequence sketches that improve genomic analysis. A current shortcoming of our method is the heuristic search for the mask variable. This challenging combinatorial problem will be an interesting avenue for future work.

## Figures and Tables

**FIG. 6.**
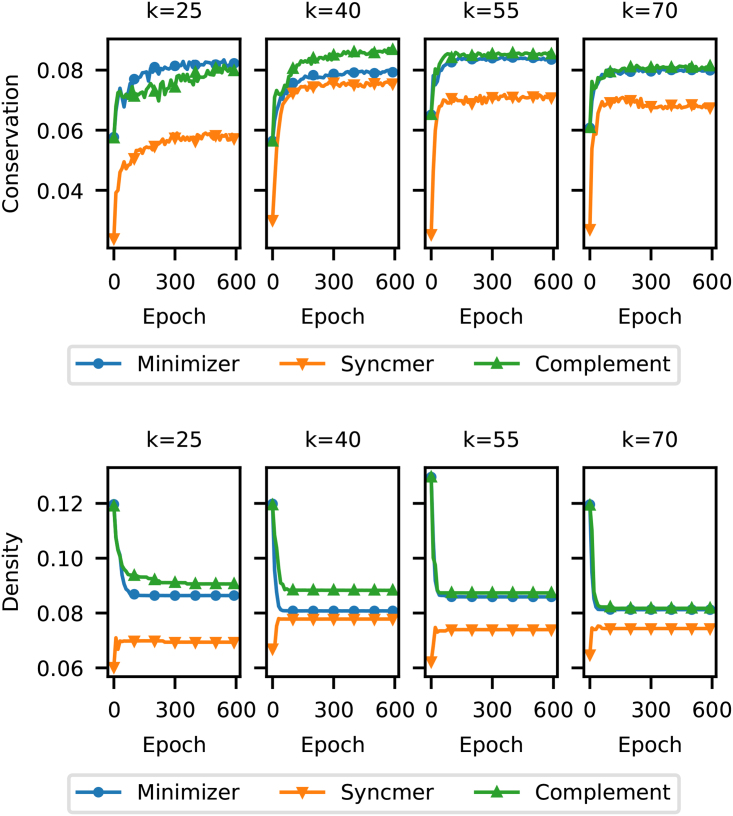
Comparing conservation and density metrics of different masked minimizers versus number of training epochs on the ChrXC sequence with w=15 and k∈{25,40,55,70}.

**FIG. 7.**
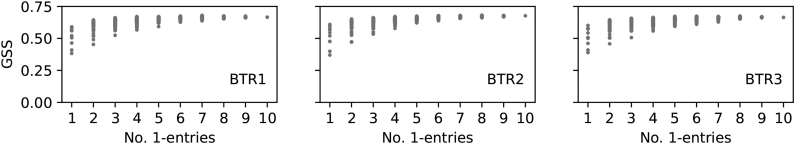
GSS versus |v| of all masked minimizers on bacterial genomes Btr1, Btr2, and Btr3.

**FIG. 8.**
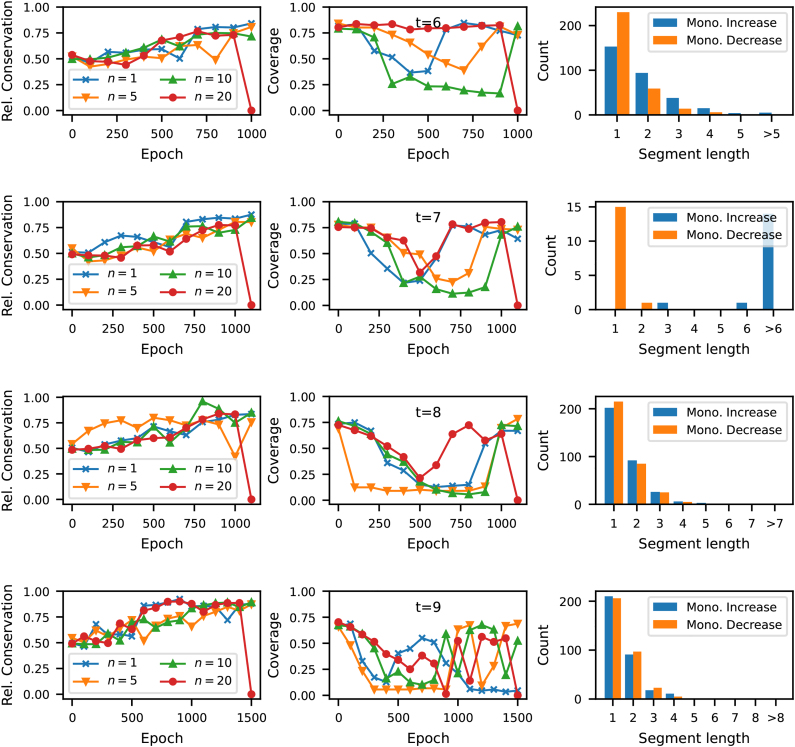
Finding the relative conservation exploit for various open syncmers using ${ℒ}_{exploit}$, w=10, and k=15 with (from top to bottom) offset t∈{6,7,8,9}.

## 8. APPENDIX

### 8.1. Other results

#### 8.1.1. Effectiveness of training on conservation and density metrics

Figure 6 demonstrates the individual effect of training the proposed loss ${ℒ}_{gss}$ on the conservation and density metrics. We observe that both the conservation and density of the open-syncmer scheme are upper-bounded by that of the minimizer scheme, which confirms the result of Corollary 1 and Corollary 2.

We observe that ${ℒ}_{gss}$ improves conservation but worsens density for the open-syncmer scheme, which is similar to our first experiment. However, this is not the case for the minimizer and complement schemes, which obtain significant improvements in both metrics over 600 training epochs (although conservation is still bounded by density at any point during the training).

This implies that our method has found a favorable trade-off between the two metrics, which, in turn, explains the sharper increases in GSS compared with that of syncmer across all experiments.

#### 8.1.2. Comparing GSS of different training losses and masks

Table 1 summarizes the result of the gradient-based methods on ChrXC. Across 18 experiments (i.e., crossing 6 settings of (w,k) with 3 loss functions), the best GSS is achieved by the minimizer mask (ℳ) on 6 experiments, the open-syncmer mask ($O_{w∕2}$) on 1 experiment, and the complement mask ($C_{w∕2}$) on 4 experiments.

**Table 1.** Comparing Generalized Sketch Score (Normalized to the Scale of 0−100) of Different Masked Minimizers with 3 Different Training Losses Across 6 Settings of (w,k) on ChrXC

(w,k)	Conservation loss (*${ℒ}_{con}$*)				Density loss (*${ℒ}_{DM}$*)				Combined loss (*${ℒ}_{gss}$*)			
	ℳ	$O_{w∕2}$	$C_{w∕2}$	V	ℳ	$O_{w∕2}$	$C_{w∕2}$	V	ℳ	$O_{w∕2}$	$C_{w∕2}$	V
10,10	70.1	65.6	68.5	70.1	71.6	52.9	72.2	74.5	$\underline{75.4}$	60.1	75.3	$\underline{75.4}$
10,15	70.6	65.0	69.6	70.6	76.8	65.6	77.9	77.9	71.9	56.6	70.4	$\underline{81.0}$
15,10	69.3	62.1	68.0	71.3	69.0	61.3	72.6	$\underline{76.5}$	68.3	59.7	70.1	75.0
15,15	71.5	66.7	71.0	$\underline{89.2}$	81.3	74.6	82.8	82.8	81.2	82.3	81.7	81.7
20,10	68.2	61.4	67.9	68.2	67.7	60.8	67.3	69.0	$\underline{72.1}$	59.5	69.0	$\underline{72.1}$
20,15	74.7	71.6	81.6	81.6	82.9	71.4	84.5	84.5	$\underline{89.8}$	79.4	89.4	$\underline{89.8}$

We, respectively, denote the minimizer mask, the open-syncmer mask, the complement mask, and the optimized mask by $ℳ,O_{w∕2},C_{w∕2},V$. The best GSS observed for each combination of (w,k) and loss function is given in bold. The best GSS for each combination of (w,k) is further underlined.

GSS, generalized sketch score.

Our optimized mask (V) achieves the best GSS in 17 out of 18 experiments, including 10 experiments where $v_{*}$ recovers either $v_{m},v_{c}$ or *v_o_*; and 7 experiments where $v_{*}$ is novel. Our loss function ${ℒ}_{gss}$ achieves the best GSS (underlined) in 4 out of 6 combinations of (w,k).

Table 2 summarizes the result of PASHA (Ekim et al., 2020), Miniception (Zheng et al., 2020), and the random ordering baseline. Generally, PASHA and Miniception outperform the random ordering baseline as expected. However, their performance is generally weaker than the gradient-based methods in Table 1 by a large margin.

**Table 2.** Comparing Generalized Sketch Score (Normalized to the Scale of 0−100) of Different Masked Minimizers Using 3 Different Discrete Construction Methods and 6 Settings of (w,k) on ChrXC

(w,k)	Miniception UHS				PASHA UHS				Random ordering			
	ℳ	$O_{w∕2}$	$C_{w∕2}$	V	ℳ	$O_{w∕2}$	$C_{w∕2}$	V	ℳ	$O_{w∕2}$	$C_{w∕2}$	V
10,10	57.4	0.0	55.1	58.2	$\underline{62.9}$	43.8	60.1	$\underline{62.9}$	26.5	21.0	28.7	28.8
10,15	47.3	25.1	48.7	50.1	75.6	19.2	$\underline{76.9}$	$\underline{76.9}$	22.2	7.9	26.2	26.5
15,10	55.7	0.0	57.6	55.7	51.0	44.4	$\underline{58.9}$	$\underline{58.9}$	27.1	27.6	25.3	27.1
15,15	43.5	36.9	47.1	50.9	52.1	30.2	55.8	$\underline{63.2}$	17.2	11.9	14.0	17.2
20,10	$\underline{60.4}$	0.0	48.9	$\underline{60.4}$	43.5	30.7	55.1	55.3	18.9	20.2	23.9	24.7
20,15	39.2	0.0	43.5	$\underline{47.6}$	32.9	31.5	39.0	39.9	13.2	9.5	12.8	13.2

We, respectively, denote the minimizer mask, the open-syncmer mask, the complement mask, and the optimized mask by $ℳ,O_{w∕2},C_{w∕2},V$. The best GSS observed for each combination of (w,k) and construction method is given in bold. The best GSS for each combination of (w,k) is further underlined.

UHS, universal hitting set.

Similar to the previous experiment, we also observe that the optimized mask (V) achieves the best GSS on 17 over 18 settings, 10 of which are clear improvements over the three baseline choices for *v*. Overall, our experiments suggest that it is beneficial to optimize *v*, and that our framework is more successful in finding sketches with high GSS than other sketch construction methods.

#### 8.1.3. Optimized masks found by our algorithm

See Table 3.

**Table 3.** Optimized Masks Found By Our Algorithm with 3 Different Training Losses Across 6 Settings of (w,k) on ChrXC, Denoted in the Format $v_{m}\setminus v_{p}$, Where *v_m_* Is the Complete Minimizer Mask, and $v_{p}$ Contains the Pruned Offsets

(w,k)	Conservation loss (*${ℒ}_{con}$*)	Density loss (*${ℒ}_{DM}$*)	Combined loss (*${ℒ}_{gss}$*)
10,10	*v_m_*	$v_{m}\setminus \left\{7\right\}$	*v_m_*
10,15	*v_m_*	$v_{m}\setminus \left\{5\right\}$	$v_{m}\setminus \left\{1,8\right\}$
15,10	$v_{m}\setminus \left\{6\right\}$	$v_{m}\setminus \left\{5,8\right\}$	$v_{m}\setminus \left\{14\right\}$
15,15	$v_{m}\setminus \left\{0,2,5\right\}$	$v_{m}\setminus \left\{7\right\}$	$v_{m}\setminus \left\{3,7\right\}$
20,10	*v_m_*	$v_{m}\setminus \left\{9\right\}$	*v_m_*
20,15	$v_{m}\setminus \left\{10\right\}$	$v_{m}\setminus \left\{10\right\}$	*v_m_*

#### 8.1.4. GSS profiles of masked minimizers on other bacterial genomes

Figure 7 shows the scatter plots of all $2^{w}-1$ masked minimizers trained on Btr1, Btr2, and Btr3 using ${ℒ}_{gss}$ with w=10 and k=15, grouped by |v|. We observe the same increasing pattern of average GSS with the size of *v*, thus confirming that the minimizer configuration is, indeed, a good default choice.

#### 8.1.5. Exploiting the relative density metric

This experiment further demonstrates that without the coverage normalization step, the conservation-density ratio (i.e., relative conservation) can be exploited. We show that this exploitative behavior can be obtained by optimizing the loss function ${ℒ}_{exploit}=\Delta \sum_{i=1}^{n}\Delta \left(P\left(S\prime_{i}\right),P\left(S\right)\right)$. This loss function differs from ${ℒ}_{con}$ by swapping the template *T* in each pairwise Δ-distance term with P(S).

The purpose of this substitution is to isolate any training signal for density (which is implicitly encoded in the template) and to directly prioritize minimizing relative conservation. As minimizers schemes must select one position per (w,k)-window by construction, they do not suffer from this exploit. We, thus, train only the open-syncmer scheme O on a random sequence with L=1000, using ${ℒ}_{exploit}$ with w=10 and k=15.

We plot the relative conservation (left-most column of Fig. 8) and coverage metrics (middle column of Fig. 8) obtained over 1000 epochs with n∈{1,5,10,20} sampled mutations per training epoch and offset t∈{6,7,8,9} (e.g., the corresponding masks are v={6},{7},{8},{9}).

We observe that ${ℒ}_{exploit}$ generally improves relative conservation as expected. However, when n=20, the optimizer finds the exploit mentioned in Section 4 after 1000−1500 epochs, which causes both metrics to become 0. The resulting sketch consequently selects no *k*-mers (i.e., 0 coverage) and is trivially conserved when mutations are introduced (i.e., infinite conservation, which is manually set to 0 in the above plots).

We further plot the number of segments with monotonically increasing or decreasing priority scores at each segment length (right most column). For every value of *t*, the exploitative solution contains no segment with more than t−1 consecutively decreasing scores.

We note that the total count for t=7 is significantly lower than other values of *t*, because the solution contains several segments of monotonically increasing scores that are relatively long, which count toward the >6 bucket. This result suggests that all lowest scoring *k*-mers are likely found within the first t−1 positions of their respective windows, and none are sub-sampled into the masked minimizer sketch.

### 8.2. Other details

See Table 4.

**Table 4.** Descriptions and Lengths of Sequences Used in Section 6

Label	Description (assembly)	Length
ChrXC	Centromere region of human chromosome X Miga et al. (2020)	3,106,132
Chr1	Human chromosome 1	233,587,144
BTR1	Blautia producta (GCA_004210255.1)	6,354,838
BTR2	Blautia hansenii DSM 20583 (GCF_002222595.2)	3,065,949
BTR3	[Clostridium] scindens (GCA_009684695.1)	3,785,527
BTR4	Blautia producta ATCC 27340 = DSM 2950 (GCA_010669205.1)	6,197,116

### 8.3. Minimizer optimization and selection methods

#### 8.3.1. Heuristic methods

A random ordering is a common heuristic choice for minimizers. The expected density of a random ordering given a window length *w* is 2∕w. Beyond this scheme, several other methods rank *k*-mers based on their frequencies in the target sequence (Chikhi et al., 2016; Jain et al., 2020) or sequentially remove k-mers from some arbitrarily constructed UHS (DeBlasio et al., 2019).

#### 8.3.2. Priority set methods

Minimizer selection schemes with expected performance guarantees are based on the theory of UHS. (Marçais et al., 2018; Orenstein et al., 2017). A (w,k)-UHS is defined as a set of *k*-mers such that every window of length *w* (from any possible sequence) contains at least one of its elements. A UHS can be thought of as a scoring function that guarantees *k*-mers in the UHS are assigned lower scores than *k*-mers outside of the UHS.

A more compact UHS has been shown to correlate with lower density, hence most UHS-based methods such as Miniception and PASHA focus on minimizing the size of UHS. Zheng et al. (2021) alternatively adopt the concept of a polar set, whose elements are sufficiently far apart on a specific target sequence, thus extending this optimization paradigm to the sequence-specific setting. The polar set optimization objective is NP-hard and currently approximated by a greedy construction (Zheng et al., 2021).

#### 8.3.3. Gradient-based method

Hoang et al. (2022a) proposed the first continuous relaxation of the discrete ordering optimization underlying the minimizer selection problem. This involves two collaborating networks: the PriorityNet focuses on constructing valid minimizer scheme (i.e., a total ordering can be reconstructed given the network), whereas the TemplateNet focuses on finding a scoring function that has a few local optima (i.e., thus implying low density).

The proposed loss function minimizes a special distance metric $\Delta_{DM}$ between the output of these networks (given *S*), and it thus induces a consensus solution that is valid and has low density on *S*. The architectures of these networks and the distance function $\Delta_{DM}$ are given in Section 8.4.

### 8.4. Parameterization of the DeepMinimizer network

#### 8.4.1. PriorityNet

The PriorityNet is parameterized by a 3-layered convolutional neural network. The first layer has filter size *k*, and subsequent layers have filter sizes 1. This design ensures that the score assigned to any *k*-mer only depends on its content. The number of hidden channels of our architecture are, respectively, 64,32, and 16 in our implementation.

#### 8.4.2. TemplateNet

The TemplateNet is parameterized by a *positional* scoring function:

In particular, *g* is a sinusoidal function modeled using a truncated Fourier series with amplitude parameters β. We refer to Hoang et al. (2022b) for more detailed explanations of this formulation.

#### 8.4.3. Distance function

The distance function proposed by Hoang et al. (2022b) is given by:

where *P*, *T* are the respective outputs of PriorityNet and TemplateNet. Compared with this formulation, our Δ function (Section 5) introduces an inner summation over the offset locations in the mask *v* to reflect the property of the masked minimizer scheme. We can equivalently express the second term in the original $\Delta_{DM}$ formulation as summing over all possible offsets (e.g., *v_m_*), and dividing by a constant factor.

The description and length of every benchmark sequence is given in Table 4. We implement our method using PyTorch and deploy all experiments on an RTX-3080 GPU. Due to limited GPU memory, each training epoch only computes the loss on a randomly sampled batch of 32 substrings of length ℓ=1500 bases. The conservation component of ${ℒ}_{gss}$ is averaged over 5 random mutations, simulated using a 10% base substitution rate. Evaluation of conservation is likewise obtained using 5 random mutations. Network weights are optimized using the ADAM optimizer (Kingma and Ba, 2014) with default parameters.
